# Celecoxib Loaded In-Situ Provesicular Powder and Its In-Vitro Cytotoxic Effect for Cancer Therapy: Fabrication, Characterization, Optimization and Pharmacokinetic Evaluation

**DOI:** 10.3390/pharmaceutics12121157

**Published:** 2020-11-28

**Authors:** Ali M. Nasr, Sameh S. Elhady, Shady A. Swidan, Noha M. Badawi

**Affiliations:** 1 Department of Pharmaceutics, Faculty of Pharmacy, Port Said University, Port Said 42526, Egypt; 2 Department of Pharmaceutics, Faculty of Pharmacy, Sinai University, Alarish, North Sinai 45511, Egypt; 3 Department of Natural Products and Alternative Medicine, Faculty of Pharmacy, King Abdulaziz University, Jeddah 21589, Saudi Arabia; ssahmed@kau.edu.sa; 4 Department of Pharmaceutics, Faculty of Pharmacy, The British University in Egypt, El-Sherouk city, Cairo 11837, Egypt; 5 The Center for Drug Research and Development (CDRD), Faculty of Pharmacy, The British University in Egypt, El-Sherouk City, Cairo 11837, Egypt

**Keywords:** celecoxib, repositioning, in-situ provesicular powder, optimization, cytotoxic assay, pharmacokinetic study

## Abstract

Introduction: Several recent studies have shown that the role of cyclooxygenase 2 (COX-2) in carcinogenesis has become more evident. It affects angiogenesis, apoptosis, and invasion, and plays a key role in the production of carcinogens. It has also been reported that COX-2 inhibitors such as celecoxib (CLX) might play an effective role in preventing cancer formation and progression. Formulation of CLX into nanovesicles is a promising technique to improve its bioavailability and anticancer efficacy. Aim: The aim of this study is to optimize and evaluate the anticancer efficacy of CLX-loaded in-situ provesicular powder composed of surfactants and fatty alcohol-based novel nanovesicles in-vitro and determine its pharmacokinetic parameters in-vivo. Methods: The novel provesicular powders were prepared by the slurry method and optimized by 3^2^ full factorial design using the desirability function. Results: Small mean particle size was achieved by the formed vesicles with value of 351.7 ± 1.76 nm and high entrapment efficacy of CLX in the formed vesicles of 97.53 ± 0.84%. Solid state characterization of the optimized formulation showed that the powder was free flowing, showed no incompatibilities between drug and excipients and showed smooth texture. The cytotoxic study of the optimized formula on HCT-116, HepG-2, A-549, PC-3 and MCF-7 cell lines showed significant increase in activity of CLX compared to its free form. The pharmacokinetic study on albino rabbits after oral administration showed significant increase in the area under the curve (AUC)_0–24 h_ and significantly higher oral relative bioavailability of the optimized formulation compared to Celebrex^®^ 100 mg market product (*p* < 0.05). Conclusion: All findings of this study suggest the potential improvement of efficacy and bioavailability of CLX when formulated in the form of in-situ provesicular powder composed of surfactants and fatty alcohol-based novel nanovesicles for its repositioned use as an anticancer agent.

## 1. Introduction

Nowadays, repositioning of drug compounds is an important topic in the pharmaceutical field, in which already existing drugs are being discovered for new therapeutic effects, in order to minimize the time and cost that is essential for drug discovery and development [1,2]. Non-steroidal anti-inflammatory drugs (NSAIDs) are drug substances that prevent the inflammation process mainly by inhibiting cyclooxygenase enzyme 2 (COX-2). COX-2 inhibition is not only associated with anti-inflammatory, antipyretic and analgesic effects but also angiogenesis, proliferation, and tumor growth [3]. Overexpression of COX-2 in a variety of cancers has been previously reported [3]. Moreover, it is reported in literature that chronic inflammation has a highly important effect on initiation and invasion of cancer [4]. It was also found that NSAIDs impede the nuclear transcription factor, NF-κB, which is found to be overexpressed in cancer cells. NF-κB modulates the expression of enzymes and proteins such as COX-2 and cyclin D1 associated with inflammation and cellular proliferation. Thus, suppressing the COX-2 enzyme may lead to a significant alleviation in the risk of cancer formation, progression, and metastasis [5]. Celecoxib (CLX) is a selective COX-2 inhibitor that belongs to the NSAIDs class that is primarily used as an anti-inflammatory drug in the treatment of conditions such as rheumatoid arthritis, osteoarthritis, or severe cases of dysmenorrhea [5]. It has also been found that CLX shows considerable anti-cancer activity in several cancer types. CLX has been proven to have anti-proliferative and anti-tumorigenic effects in vitro and in vivo for a number of different organ specific cancers including breast, prostate, lung and liver cancers [6]. It introduces a new approach for preventing cancer formation and for improving efficacy or further eluding drug resistance alone or in combination with chemotherapy [5]. CLX is classified according to the Biopharmaceutical Classification System (BSC) as a BCS class II substance (pKa = 11.1) due to its low aqueous solubility and good permeability, resulting in low bioavailability, high variability in drug absorption and blood levels after its oral administration [7]. Therefore, administration of CLX in a colloidal drug carrier system could be undertaken in order to overcome these side effects. Incorporation of CLX into the drug delivery system is used for enhancing its bioavailability, especially in cancer therapy, through improving the tissue distribution and then, facilitating internalization of drug carrier systems by tumor cells [5]. Several nanosystems have been stated to enhance the anticancer activity of drugs, due to their ability to improve cellular uptake because of their small size [8]. Vesicular systems such as liposomes and niosomes are the most common carriers used in order to entrap the drug molecules [9]. Regrettably, these vesicular systems have some drawbacks such as limited encapsulation, stability, and scaling up obstacles, which provoke the need for evolving new vesicular systems that can overcome such problems [10]. Among the promising new vesicular systems are in-situ forming provesicular drug carriers, which are pro-nanovesicles consisting of dry free flowing powder coated with surfactant, which can be hydrated just before use resulting in the formation of the nanovesicles facilitating the absorption of the incorporated drugs [11,12]. In addition, pro-nanovesicles can be formulated in a single-step that in turn converts to real nanovesicles after administration, for example proliposomes and proniosomes, unlike other nanovesicles that needs at least two steps; preparation of the dispersion then solidification or drying, which is a time-consuming and difficult procedure [13]. Moreover, due to them being surfactant-based vesicles without aqueous media, they can overcome the stability problems accompanied with many nanoparticles, which are mainly drug leakage, fusion, and aggregation [14]. It has also been found that they can enhance the bioavailability of absorbed compounds by facilitating transcellular and paracellular absorption [12]. The only disadvantage of these novel nanovesicles is linked to all other carrier-based nanoforms, that is, the dependence of the efficiency of the nanoform on the quality of the carrier, in this study—the spray-dried lactose. According to Healy et al., the efficiency of lactose-based powder formulation is highly dependent on the quality of lactose, its source, particle size and particle size distribution and fine-lactose content [15]. However, this might be of greater importance in routes other than the oral route such as the inhalation. One of the simplest, most rapid, and efficient methods of preparing free flowing powder-based nanoparticles is the slurry method. This method involves preparation of a slurry in a round bottom flask using carrier, fatty alcohol, and surfactant solution. The least amount of organic solvent can be added to obtain slurry. The slurry is dried by applying a vacuum to completely evaporate the organic solvent and obtain free-flowing powder [14]. It is assumed that encapsulation of CLX into novel fatty alcohol, non-ionic surfactant-based, in-situ forming provesicular powder could improve its anticancer activity as well as its bioavailability. Various vesicle-forming non-ionic surfactants can be used such as sorbitan monopalmitate (span 40) and sorbitan monostearate (span 60). Different fatty alcohols such as cetyl alcohol can be used as a stabilizer for the nanovesicles formed upon hydration [16]. Therefore, the aim of this study was to develop, evaluate and optimize CLX-loaded cetyl alcohol/sorbitan monopalmitate in-situ provesicular powder (CLX-IPP). The CLX-IPP was prepared using the slurry method according to 2^3^ full factorial experimental design in order to detect the effect of formulation variables on the prepared nanovesicles using Design-Expert^®^ software to select the optimum formula. Afterwards, the anti-proliferative effect of the CLX-IPP optimized formula was evaluated in-vitro in human hepatocellular cancer (HepG-2), human lung carcinoma (A-549), human colon cancer (HCT-116), human breast cancer (MCF-7) as well as human prostate cancer (PC-3) cancer cell lines and then compared to free CLX. Finally, a pharmacokinetic study was conducted to evaluate the bioavailability of CLX-IPP optimized formula in comparison to CLX market capsules.

## 2. Materials and Methods

### 2.1. Materials

Celecoxib was a kind gift from Memphis Cairo, Egypt. Cetyl alcohol was obtained from Sigma-Aldrich, Germany. Sorbitan monopalmitate (span 40) was purchased from Oxford Laboratory Chemicals, Mumbai, India. Spray-dried lactose was supplied by Medical Union Pharmaceuticals, Ismailia, Egypt. Chloroform and methanol were purchased from Fisons Scientific Equipment, Glasgow, UK. Dimethyl sulfoxide (DMSO), 3-(4,5-dimethylthiazol-2-yl)-2,5-diphenyl-2H-tetrazolium bromide MTT and trypan blue dye were purchased from Sigma, St. Louis, MO, USA. Fetal bovine serum, Dulbecco’s Modified Eagle’s medium (DMEM), Roswell Park Memorial Institute medium (RPMI)-1640, N-(-2-Hydroxyethyl)piperazine-N’-(2-ethanesulfonic acid) (HEPES) buffer solution, l-glutamine, gentamycin and 0.25% trypsin-EDTA were purchased from Lonza, Verviers, Belgium. All other solvents and chemicals used were of analytical grade and were used as received without further modifications.

### 2.2. Methods

#### 2.2.1. Systematic Design of Experiments

A complete 3^2^ factorial design was applied for the preparation and optimization of CLX-loaded nanovesicles using Design-Expert 11 program (Stat-Ease, Inc., Minneapolis, MN, USA). Here, we considered two independent variables of this model, which include amount of lipid (X1), and quantity of surfactant employed (X2). The outcome measures for this design included vesicle size (Y1) and entrapment efficiency % (Y2). Table 1 lists the independent and dependent variables used in our study with their levels and desirability constraints. Optimization of all variables was completed using the desirability function firstly introduced by Derringer and Suich [17]. The desirability function is based on the concept that the quality of a novel formulation developed that has many features is totally unacceptable if even one of them is outside of a desirable limit. The desirability function is used to corroborate the compliance with the criteria selected for all involved responses and to provide the best value of compromise in the desirable joint response [18]. This can be achieved by converting the multiple responses evaluated into a single one, combining the individual responses into a composite function followed by its optimization. The formulation with the highest desirability function value is selected for further investigations.

#### 2.2.2. Preparation of CLX-Loaded In-Situ Provesicular Powders

The provesicular CLX-loaded powder was prepared according to the slurry method approach. Some minor modifications were made to the method described by Khan et al. [19]. Precisely weighed amounts of cetyl alcohol, span 40 and CLX were dissolved in 10 mL of organic solvent (methanol and chloroform in ratio 3:7, respectively) as shown in Table 2. Spray-dried lactose was added to the previous solubilized mixture in a 100 mL rotary-evaporator flask. The solvent mixture was then evaporated using Heidolph rotary evaporator (P/N Hei-AP Precision ML/G3, Schwabach, Germany). The temperature of the water bath was kept at 45 ± 2 ºC. The speed of rotation was adjusted at 60 rpm. A vacuum was applied under a pressure of 600 mmHg until the solvent was completely evaporated and a thin powder film had formed on the wall of round flask. The resulted dry powder was well scratched from the flask wall, removed, and finally dried overnight at room temperature to obtain free-flowing dry product [20]. Finally, the dried formulations were kept in well closed containers in a cooler kept at 4 °C for further characterization.

#### 2.2.3. Formation of CLX-Loaded Nanovesicles

The formed dried formulations were transferred to nanovesicles by hydrating with 10 mL of phosphate-buffered solution, pH 7.4 at 37 °C ± 1 °C with gentle vortexing for 2 min using Thermolyne Vortex Mixer (Thermo Scientific, Maxi-Mix II, 120 V, 50/60 Hz, Austin, TX, USA).

#### 2.2.4. Characterization of the Prepared CLX-Loaded Nanovesicles Formulations

##### Vesicle Size (PS), Polydispersity Index (PDI) and Surface Charge (ZP) Determination

Freshly prepared hydrated formulation (1 mL) was diluted with 10 mL of phosphate-buffered solution, pH 7.4 at 37 °C ± 1 °C by vortexing to produce a homogenous dispersion with a measurable scattering intensity. The formed dispersion was then used to measure vesicle size, zeta potential and size distribution using Malvern Zetasizer (Nano ZS, Malvern Instruments Ltd., Malvern, UK). All determinations were performed at room temperature (25 °C) in triplicates. All values were reported as the mean and its standard deviation [21].

##### Determination of CLX Entrapment Efficiency (EE %) in the Prepared Nanovesicles

Entrapment efficiency % was calculated by an indirect method after the hydration of the CLX-IPP powders. Exactly, weighed CLX-IPP powder was dispersed in phosphate buffer pH 7.4 and vortexed for 2 min. Then, the CLX-IPP nanovesicles dispersion was centrifuged at 12,000 rpm and 4 °C for 1 h using a cooling centrifuge (2-16KL, Sigma Laborzentrifugen GmbH, Osterode am Harz, Germany); the supernatant was then withdrawn and the amount of unentrapped CLX was determined at 256 nm [22] using a UV spectrophotometer (V-630, Jasco, Tokyo, Japan) [12]. The EE % was calculated using the following equation:

(1) $$\mathrm{EE}\;\%\;=\frac{\mathrm{Total}\;\mathrm{amount}\;\mathrm{of}\;\mathrm{CLX}\;-\mathrm{amount}\;\mathrm{of}\;\mathrm{CLX}\;\mathrm{in}\;\mathrm{supernatent}}{\mathrm{Total}\;\mathrm{amount}\;\mathrm{of}\;\mathrm{CLX}}\times 100$$

All measurements were performed in triplicate and the mean values of EE % were plotted together with standard deviation.

##### Optimization of Formulation Variables

The target of optimization was to look down on the levels of the variables under study to produce a formulation with the highest quality. The factorial designs are utilized for identifying the factors that might influence the characteristics of a novel drug delivery system [23]. The prepared CLX nanovesicles were optimized for the responses Y1 (PS) and Y2 (EE %). The aim of the optimization design is to maximize EE % and to minimize PS. Based on illustrations from the response surface plots, the optimized formulation was chosen by assigning desired goals to the response variables. The optimized formulation selected was subjected to a series of extensive in-vitro and in-vivo studies.

#### 2.2.5. Micromeritic Properties of the Optimized CLX-Loaded Provesicular Powder Formulation

The flow properties of powder are vital in handling and processing operations. The flow behavior of CLX-loaded provesicular powder formulation was evaluated by measuring: angle of repose, Carr’s compressibility index and Hausner ratio [24]. The angle of repose was assessed by the fixed funnel approach. Carr’s index and Hausner ratio were measured by determination of the bulk and true density of the powder according to the following equations [25]:

(2) $$\mathrm{Carr}’s\;\mathrm{Compressibility}\;\mathrm{index}\;=\;\frac{\rho t-\rho b}{\rho t}\times 100$$

(3) $$\mathrm{Hausner}\;\mathrm{ratio}=\frac{\rho t}{\rho b}$$

where *ρb* and *ρt* are the bulk density and true density, respectively.

#### 2.2.6. Solid State Characterization of the Prepared Optimized Formulation

##### Thermal Analysis Using Differential Scanning Calorimetry (DSC)

The molecular state of the drug in the optimized prepared provesicular powder was evaluated using differential scanning calorimetry (DSC). This analysis also provided a useful framework for examining the crystallinity and the physical nature of CLX in the optimized formulation. Samples of pure CLX powder, cetyl alcohol, span 40, and the physical mixture of all previous components in addition to the optimized CLX formulation were scanned using differential scanning calorimeter (DSC 6000; Perkin Elmer, Waltham, MA, USA). Analysis was performed by heating 5 mg of each sample individually in a sealed aluminum pan, which was then covered with an aluminum cover. All samples were scanned over the temperature range from ambient temperature to 350 °C at a scanning rate of 10 °C/min under nitrogen purge at 30 mL/min. The reference substance used in the analysis was pure indium (In). The resultant thermograms were compared and analyzed [26].

##### Fourier Transform Infrared (FT-IR) Spectroscopy

Infrared spectra of CLX, span 40, cetyl alcohol, physical mixture, plain formula, and medicated optimized powder formulation were obtained using the FT-IR spectrophotometer VERTEX 70 (Bruker Corporation, Ettlingen, Germany). The FT-IR spectrophotometer is coupled with Platinum Diamond attenuated total reflectance (ATR), which consists of a diamond disc as an internal reflection element. The spectrum of each sample was measured in the range of 4000 to 500 cm^−1^ wavenumber region. The powdered sample was placed on the ATR crystal, and then the spectrum was recorded. Before each sample analysis, the background used was the spectrum of air. The spectra of samples as well as the background were taken in a room with a temperature 22–24 °C, at a spectral resolution of 4 cm^−1^. For each measurement, 32 scans were performed.

##### Surface Characteristics of the Optimized CLX-Loaded Provesicular Powder Using Scanning Electron Microscopy Analysis

Scanning electron microscopy (SEM) analysis was performed on the powder of optimized formulation to evaluate the morphological characteristics of the powder. The sample was sprinkled and fixed on a SEM holder with double sided adhesive tape and coated with a layer of gold of 150 °A for 2 min using a sputter coater (Edwards, S-150A, Cambridge, UK) working in a vacuum of (3 × 10^−1^ atm) of argon gas. The sample was examined using a scanning electron microscope (Jeol, JSM T20, Tokyo, Japan) at 15 kV acceleration voltage at room temperature.

#### 2.2.7. Morphology of the Optimized CLX-Loaded Nanovesicles Using Transmission Electron Microscopy

The morphology of the prepared vesicles after hydration of the powder of the optimized CLX-IPP formulation was investigated using the transmission electron microscopy (TEM) technique. Simply, a single sample drop was diluted 10 times using deionized water then one drop of this diluted dispersion was applied to a collodion-coated 300 mesh copper grid to allow some of the vesicles of the CLX-IPP to adhere to collodion (pure grade that meets analytical specification of Ph Helv VIand DAB6, Fluka Chemie GmbH, Buchs, Schweiz) and left for 5 min. The excess remaining nanovesicular dispersion was removed by adsorbing the drop with the tip of a piece of filter paper (Whatman International Ltd., Maidstone, England). A drop of two percent aqueous solution of uranyl acetate was applied on the sample on the copper grid for 1 min. The residual solution was then swept away, and the sample was dried by air and examined with the TEM (Jeol, 1200 EXII, Tokyo, Japan) at 74 kV. The obtained TEM image was analyzed for size distribution by the software Nano Measurer 1.2.5 (Fudan University, Shanghai, China).

#### 2.2.8. In-Vitro Release of CLX from the Optimized Formulation

The in-vitro release behavior of the CLX-IPP optimized formula was carried out using the dialysis bag method and compared with free CLX. A certain weight of CLX-IPP powder and also the same weight of free CLX were resuspended in phosphate-buffered pH 7.4 and vortexed for 2 min. The bags were primarily soaked in phosphate-buffered pH 7.4 for 24 h before being used. Accurately, 1 mL of each sample (CLX-IPP or free CLX) was transferred into the dialysis bag (cellulose membrane dialysis bag, with a molecular weight cut off of 12,000 Da, Sigma-Aldrich, St. Louis, MO, USA) representing the donor compartment. The dialysis bag was then placed into 20 mL phosphate-buffered pH 7.4 containing 1% (*w*/*v*) sodium lauryl sulfate (SLS), acting as the receptor compartment, with the temperature maintained at 37 ± 0.5 °C in an incubation shaking water bath (WSB-18, Daihan Scientific Co. Ltd., Gangwon, South Korea) and shaked at 50 rpm. Samples were withdrawn at 1, 2, 3, 4, 5, 6, 7, 8, 12, 18 and 24 h time intervals, replaced with fresh buffer to keep the sink condition and then analyzed spectrophotometrically at 256 nm [10,27,28].

#### 2.2.9. In-Vitro Cytotoxicity Study of the CLX-IPP Optimized Formula Using MTT-Assay

In this study, cytotoxicity evaluation against five different cancer cell lines was undertaken for CLX-IPP optimized formula in addition to the free CLX. The concentrations that induced 50% growth inhibition as a result of treatment with CLX-IPP optimized formula and the free CLX were obtained and compared.

##### Cell Lines and Cell Culture

Five chosen cancer cell lines, namely human hepatocellular cancer (HepG-2 cells), human lung carcinoma (A-549 cells), human colon cancer (HCT-116 cells), human breast cancer (MCF-7 cells) and human prostate cancer (PC-3 cells), were obtained from the American Type Culture Collection (ATCC, Rockville, MD, USA). Cells were cultured in Roswell Park Memorial Institute medium (RPMI-1640) and supplemented with 10% fetal bovine serum in addition to gentamycin (50 µg/mL). The cells were kept in a humidified atmosphere of 5% CO_2_ at 37 °C and sub-cultured from two to three times per week. 

##### Antiproliferative Activity (MTT-Assay) 

The growing cells from different tumor cell lines were seeded for 24 h in Corning^®^ 96-well tissue culture plates (5 × 10^4^ cell/well) for attachment. Thereafter, cells were treated with CLX-IPP optimized formula and the free CLX to accomplish ten concentrations (1 µg/mL to 500 µg/mL) of each sample and then incubated for 48 h using vehicle (0.5% DMSO) as a control. In brief, the treated cells’ viability was detected by a MTT test in this technique, the medium was discarded and replaced with 100 µL of fresh culture RPMI-1640 medium then 10 µL of the 12 mM MTT stock solution (5 mg of MTT in 1 mL of PBS). Afterward, cells were kept at 37 °C and 5% CO_2_ for 4 h then 85 µL aliquot of the medium was discarded from the wells and 50 µL of DMSO was added and mixed followed by a 10 min incubation period at 37 °C. Using a microplate reader (SunRise, TECAN, Inc, Morrisville, NC, USA), absorbance was measured at 590 nm. Percentage cell viability was calculated in treated cells against that of control. Half-maximal inhibitory concentration (IC50) was determined using Graphpad Prism 5 software (San Diego, CA, USA) [29].

#### 2.2.10. Pharmacokinetic Study of CLX in Rabbits

##### The Design of the Pharmacokinetic Study

The in-vivo pharmacokinetic study was performed on twelve white rabbits (the weights were 2–2.5 kg and provided by the animal laboratory, Faculty of Pharmacy, The British University in Egypt). The rabbits were randomly divided into two groups—six rabbits in each group—and were housed individually in stainless steel cage with free access to food and water and kept conscious throughout the whole experiment. The room was maintained in a 12 h dark/light cycle under 24 ± 1 °C and 55 ± 5% relative humidity. On the day before the experiment, all rabbits were fastened overnight for 12 h. Through the oral route, group one was administered powdered Celebrex^®^ (100 mg) capsule suspension, and the other was administered the hydrated powder dispersion of the optimized formula, both in the same dose equivalent to 10 mg/kg body weight CLX. At predetermined time intervals (0, 0.5, 1, 2, 3, 4, 6, 8 and 24 h), two milliliter blood samples were withdrawn from the ear vein. Collected blood samples were centrifuged at 10,000× *g* for 15 min using a tabletop centrifuge to obtain plasma samples, the obtained plasma samples were stored at −80 °C until they were assayed by the validated HPLC procedure mentioned below. The in-vivo pharmacokinetic study was carried out according to the guidelines approved by the ethics committee of Faculty of Pharmacy, The British University in Egypt, approval number Ex-2007 (approval date: October 2020).

##### Drug Assay in Plasma

The HPLC method was completed according to Mamidi et al., with slight modifications [30]. Aliquots of rabbit blood collected during the pharmacokinetic studies were immediately centrifuged to obtain plasma and were extracted with dichloromethane/ethyl acetate (1:1, *v*/*v*) mixture. After evaporation of the organic solvent, the residue was reconstituted in the mobile phase and analyzed by the HPLC instrument (Hitachi LaChrome Elite, Tokyo, Japan). The column used was a 250 × 4.6 mm (i.d.), 5 µm Inertsil ODS-2 column (Gl Sciences, Tokyo, Japan). The mobile phase (35% of 0.01 M KH_2_PO_4_ buffer (pH 3.2): 65% of acetonitrile mixture, *v*/*v*) was delivered at 1 mL/min. Quantitation was achieved with UV detection at 255 nm. The HPLC was operated by EZchrom Elite version 3.3.2 SP1 by Agilent.

##### Pharmacokinetic Analysis

The average of pharmacokinetic parameters of CLX was determined for all rabbits in both groups using pharmacokinetic software version 5.2 (PK function for Microsoft Excel, Pharsight Corporation, Sunnyvale, CA, USA). Analysis of data of CLX plasma concentration against the time curve for the 24 h following the oral intake of both optimized CLX-IPP formulation and the powder of market product capsules groups were performed using non-compartmental analysis. The time taken to reach the maximum plasma concentration (tmax), half-life time (t_1/2_), peak plasma concentration (Cmax), the area under the curve (AUC_0–24_ h), and the mean residence time (MRT) was determined. The relative oral bioavailability of the optimized CLX-IPP formulation and the market product was calculated using the following Equation (4):

(4) $$\mathrm{Relative}\;\mathrm{bioavailability}\;=\;\frac{AUC\;\mathrm{of}\;\mathrm{the}\;\mathrm{optimized}\;\mathrm{formula}}{AUC\;\mathrm{of}\;\mathrm{the}\;\mathrm{market}\;\mathrm{product}}\times 100$$

#### 2.2.11. Statistical Analysis

The data were statistically analyzed using the software SPSS 11.0 (SPSS Inc., Chicago, IL, USA). A minimum *p*-value of 0.05 was used as the significance level for all tests. Unpaired Student *t*-test was used for the analysis of the data obtained from the pharmacokinetic study of untransformed data for the pharmacokinetic parameters C_max_, t_1/2_, AUC_0–24_ h. Data are reported as mean ± S.D.

## 3. Results and Discussion

### 3.1. Effect of Formulation Variables on the PS of the Prepared CLX Nanovesicles

**Figure 1:**
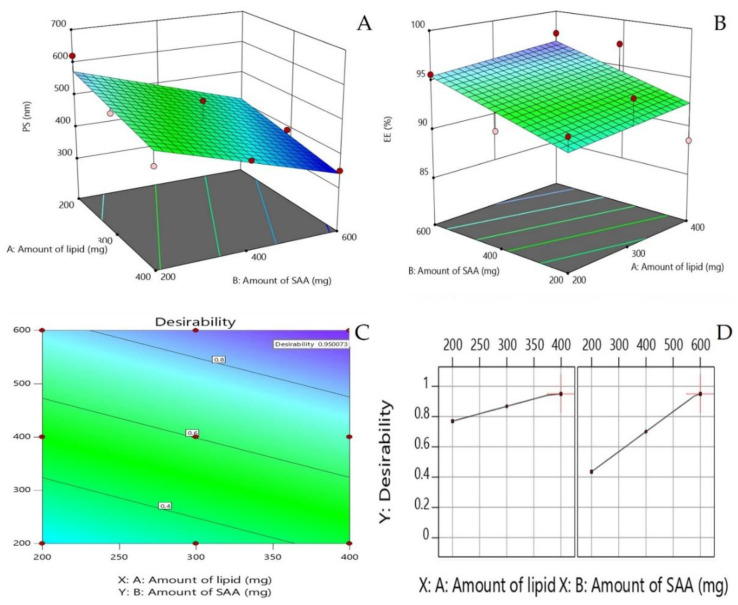
Response 3D plot for the effect of formulation variables: amount of lipid, and surfactant amount on (**A**) the mean particle size of prepared celecoxib (CLX) nanovesicles Y1, (**B**) the entrapment efficiency Y2, (**C**) the contour plot of the desirability function and (**D**) the effect of formulation variables on desirability.

It is well reported that the particle size of drug delivery carriers has a great effect on pharmacokinetics, tissue distribution and clearance. Certain physiological processes such as hepatic uptake and accumulation, tissue diffusion, tissue extravasation and kidney excretion significantly depend on particle size [31]. One of the recommended ways to enhance the oral bioavailability of CLX is to develop nanovesicles with optimum particle size. Results illustrated in Table 3 show that particle size values ranged between 351.7 ± 1.76 and 621.2 ± 2.82 nm. The data indicate the surface-weighted mean diameter of the prepared formulations. The effect of the amount of lipid (X1), and surfactant amount employed (X2) on the particle size of the prepared CLX nanovesicles is graphically interpreted as a response 3D plot as shown in Figure 1A. Only the amount of span 40 (X2) significantly influenced the PS of the nanovesicles. It was clear that the mean PS was significantly decreased by increasing span 40 amount. The lowest PS records were observed in formulations with the highest amount of span 40; formulation F9 (600 mg span 40, 351.7 ± 1.76 nm) and the largest PS values were remarked in formulations with the lowest amount of span 40; formulation F1 (200 mg span 40, 621.2 ± 2.82 nm). Increasing amount of span 40 might lead to an increase in the surface activity, forming nanovesicles with smaller PS. Surfactant concentration also affects the final size of the particles. High surfactant concentration decreases surface tension and stabilizes newly developed surfaces during the formulation of nanoparticles [32]. However, low insufficient quantity of surface active agent in lipid-based nanocarriers causes instability [33]. This observation agrees with findings observed by Negi et al. who studied the impact of surfactant concentration on the vesicle size of venlafaxine niosomes [34]. Additionally, a similar pattern of results was observed in the study of Elsayed et al., who stated that vesicle size decreased with increasing the surfactant concentration [13]. The increase in the chain length of fatty alcohols leads to an increase in hydrophobicity, contributing to lower hydrophilic lipophilic balance (HLB) and a higher critical packing parameter, which could increase the particle size. According to Vankayala et al., who tested different types of fatty alcohols as lipid components of surfactant/fatty alcohol vesicles, the particle size increased significantly (*p* < 0.05) with increase in the ratio of fatty alcohol to surfactant. They attributed this to increasing the stabilizing effect due to the higher concentration of fatty alcohol as a stabilizer in the vesicles.

With respect to the PDI, a value of 0 specifies a wholly monodispersed particle population, while a value of 1 recommends highly polydispersed vesicles [35]. The PDI of all prepared formulations ranged between 0.302 ± 0.046 (F9) and 0.494 ± 0.019 (F1), revealing uniform size distribution and good homogeneity (Table 3). It was observed that formulations with smallest values of PDI (F9) contain highest amount of span 40 (600 mg). This formulation (F9) also showed the smallest particle size record.

### 3.2. Effect of Formulation Variables on ZP of the Prepared CLX Nanovesicles

Zeta potential is the measure of total charges acquired by particles. It is of high importance to obtain accurate judgments about the stability of colloidal dispersions. Generally, the colloidal dispersion is considered stable when the zeta potential (ZP) value is around 30 mV due to electrostatic repulsion between particles [36]. For nanoparticles, a high value of zeta potential will ensure system stability, which causes a system to resist agglomeration. When zeta potential is low, attraction exceeds repulsion, and the dispersion will flocculate. Therefore, colloidal dispersions with high zeta potential (either negative or positive) are electrically stabilized [37]. In this study, the values obtained for zeta potential were in the range between −36.1 ± 0.67 and −41.3 ± 0.9021 mV as seen in Table 3. Although the vesicles were composed of non-ionic amphiphiles, and thus not expected to possess high surface charge, they have relatively high zeta potential values. Vankayala and coworkers presented an explanation, suggesting that high surface charge values might be credited to adsorption of hydroxyl ions onto the surface of vesicles from the fatty alcohol, i.e., hydroxylation of surfactants occurred when they came in contact with the aqueous media [16]. These findings indicate that the prepared vesicles have sufficient charges that would prevent their coalescence. All formulations showed high surface stability and are considered stable. Being in the form of in-situ provesicular powder, the storage of the formulations in the solid state offers long term stability. Our findings present proof that the prepared novel form is not only stable when dry, but also the formed nanovesicles show high stability and low probability of co-aggregation.

### 3.3. Effect of Formulation Variables on EE % of the Prepared CLX Nanovesicles 

The EE % of CLX in the prepared nanovesicles ranged between 88.56 ± 0.95% and 97.53 ± 0.84%. Table 3 and Figure 1B show the percentage of EE % of the prepared CLX-loaded nanovesicles. As shown in the figure, higher EE % was achieved by increasing the surfactant concentration. Kumar and Rajeshwarrao mentioned that by increasing the surfactant/lipid level, the amount of drug to be encapsulated also increases [38]. Elsayed et al. prepared similar cetyl alcohol-based vesicles and found that increasing the surfactant amount resulted in higher EE % of rosuvastatin in the prepared in-situ forming nanovesicles [13]. Higher EE % values were obtained when increasing the surfactant concentration relative to the phospholipids in prepared sofosbuvir-loaded bilosomes [39]. All these data suggest that increased surfactant concentration increases the solubilization of CLX in fatty alcohol, resulting in higher EE values. Span 40 has a low HLB value and is characterized by a long alkyl chain and it has been reported that increasing the alkyl chain of surfactants in surfactant-based vesicles results in higher EE % of hydrophobic drugs in the prepared nanovesicles [40,41].

### 3.4. Optimization of Formulation Variables

The optimization was completed based on 3^2^ full factorial design. Based on this design, the two responses were simultaneously optimized using the linear scale desirability function. This statistical function aims to find operating conditions or formulation proportions that ensure compliance with the criteria of all the involved responses. It seeks the most favorable and compromising point in the design space that fulfils the goal for each dependent variable [42]. Desirability constraints (goals) of the two evaluated responses—mean particle size and EE %—are shown in Table 1. Zeta potential was not selected as an optimization response as all formulations achieved high zeta potential values and proved high stability. The small difference between the potential values does not reflect a crucial disadvantage in any formulation prepared. The most suitable model for fitting Y1 and Y2 values was the linear one. From Figure 1C,D, the formulation variables’ values that best achieved the response goals were the highest lipid amount and the highest surfactant amount, which was achieved in formulation F9. High desirability function was achieved by this optimized formulation of value 0.95. This high desirability value indicates that the highest cetyl alcohol amount and highest span 40 amount achieved smallest mean particle size and highest entrapment efficiency collectively. F9 is the CLX-IPP optimized formulation and it was extensively studied, characterized, and evaluated for cytotoxic effect on different cancer cell lines. 

### 3.5. Micromeritic Properties of the Optimized CLX-Loaded Provesicular Powder Formulation

The flow properties of the optimized CLX-IPP were evaluated by determination of the angle of repose, Carr’s compressibility index and Hausner ratio. All micromeritic values showed that the powder was free flowing with excellent flowing properties. The angle of repose was 26.76° ± 0.68. It has been reported that the angle of repose being smaller than 30° indicates excellent flow characteristics of the powder, while high values of the angle of repose are obtained when the particles are of high cohesion or high internal friction [43]. Carr’s compressibility index value was found to be 19.00% ± 3.79, while Hausner ratio was 1.24 ± 0.06. The excellent flow properties were due to the use of spray-dried lactose as a carrier for the provesicles, which is advantageous for better flow and filling during manufacture.

Particles with high internal friction or cohesion increase the value of the angle of repose.

### 3.6. Solid State Characterization of the Prepared Optimized Formulation

#### 3.6.1. Thermal Analysis Using Differential Scanning Calorimetry (DSC)

**Figure 2:**
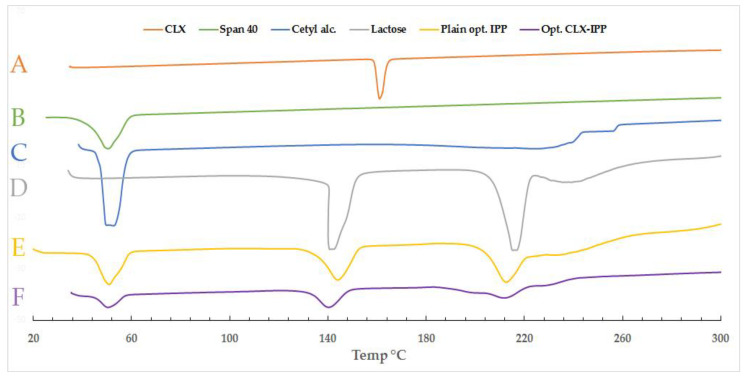
Differential scanning calorimetry (DSC) thermograms of (A) pure celecoxib (CLX), (B) span 40, (C) cetyl alcohol, (D) lactose, (E) plain optimized formula (plain opt. in-situ provesicular powder (IPP)) and (F) celecoxib-loaded optimized formula (opt. CLX-IPP).

DSC is an important technique used to confirm either the drug is of crystalline or amorphous nature within the prepared powdered formula. It also elucidates all possible interactions of the active ingredient with other ingredients such as the carrier or the vesicle-forming substances. The DSC thermograms of CLX, span 40, and cetyl alcohol, spray-dried lactose are shown in Figure 2. The figure also shows the thermograms of the drug-free optimized formula as well as the medicated optimized CLX-IPP. As seen from the figure, the CLX showed a melting endothermic peak at 161.02 °C. This value was found in between the sharp peaks characteristic for the CLX powder as mentioned by Fouad et al., and Homayouni et al., who stated that the peak of pure CLX was 159.49 °C and 165.51 °C, respectively [44,45]. The heat of fusion was found to be −297.08 mJ and the DSC curve of CLX powder revealed typical behavior of a crystalline anhydrous substance. The span 40 and cetyl alcohol, which form the nanovesicle after hydration, show sharp endothermic peaks at a similar temperature region at 50.5 °C and 49.71 °C, relatively. The heat of the endothermic peaks for cetyl alcohol was −1474.52 mJ, while spray-dried lactose peaks were −1224.36 mJ and −1604.50 mJ. Lactose powder showed two characteristic peaks, a desolvation endotherm is evident at 141.19 °C region, and a melting endotherm at approximately 216.74 °C with heat of −1224.36 mJ and −1604.50 mJ for the two peaks, respectively. Huang et al. and Al-Akayleh et al. mentioned the same approximate temperature values of desolvation and melting peaks for a spray-dried lactose thermogram [46,47]. Both plain and drug-loaded optimized formulations showed three peaks in the same regions. The plain IPP formula showed peaks at 50.77 °C, 143.97 °C and 212.47 °C, respectively, while in the thermogram of the optimized CLX-IPP formulation, the three peaks were at 50.38 °C, 148.31 °C and 211.88 °C. The three peaks are in the same regions of all ingredients of IPP, while there was a complete absence of peaks in the region where the CLX peak was expected to appear. The absence of a conspicuous peak over the range of 150–170 °C in the optimized CLX-IPP powder might be a good indication of the conversion of the native crystalline form of the drug to a molecular or amorphous state when dispersed in the vesicle-forming ingredients. The transformation of CLX from crystalline to an amorphous or molecularly dispersed form is beneficial for enhancing the dissolution and release as an amorphous form of the drug does not require energy to break up the crystalline lattice [48].

#### 3.6.2. Fourier Transform Infrared (FT-IR) Spectroscopy

**Figure 3:**
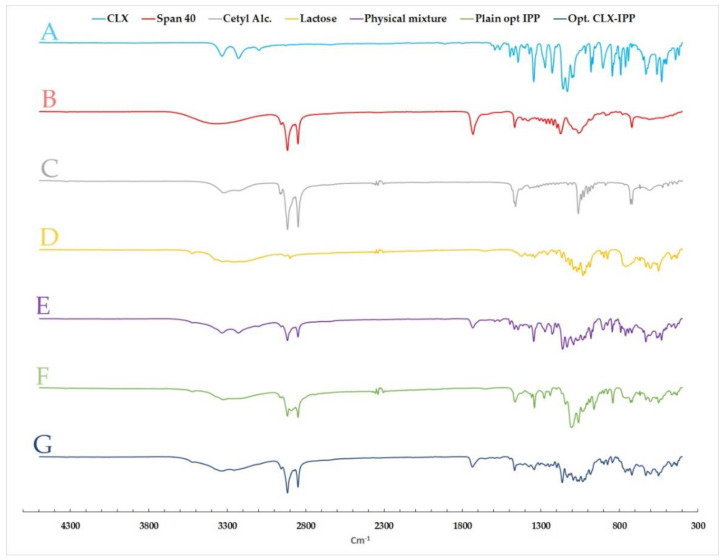
FT-IR spectra of (A) pure celecoxib (CLX), (B) span 40, (C) cetyl alcohol, (D) lactose, (E) physical mixture, (F) plain optimized formula (plain opt. IPP) and (G) celecoxib-loaded optimized formula (opt. CLX-IPP).

FTIR spectra of CLX, span 40, cetyl alcohol, lactose, physical mixture, plain formula and the optimized formula were evaluated to study the computability between the drug with other excipients (vesicle-forming substances) as seen in Figure 3. Pure CLX indicated characteristic bands at 3333.13 and 3228.15 cm^−1^, which are assigned to SO_2_NH_2_ stretching vibrations [49]. The FTIR spectra of span 40 showed a characteristic broad peak of O–H stretching at 3366 cm^−1^ as well as another peak at 1732 cm^−1^ of C=O stretching (ester) [50]. While cetyl alcohol revealed bands at 3322, 2915 and 2849 cm^−1^ of O-H stretch, CH stretch and CH_2_, respectively [51]. Moreover, lactose spectra presented a broad peak at 3258 cm^−1^ of O-H stretching [52]. Regarding the physical mixture, there was no appearance or disappearance of drug or other excipients peaks, which confirmed the compatibility between all formulation ingredients. It was also observed that all peaks found in plain and loaded nanovesicles (optimized formula) resulted from the superposition of their separated FTIR spectra. The characteristic peaks of CLX in the optimized formula FTIR spectra were not present, which proved successful encapsulation of CLX in the optimized CLX-IPP formulation.

#### 3.6.3. Surface Characteristics of the Optimized CLX-Loaded Provesicular Powder Using SEM

**Figure 4:**
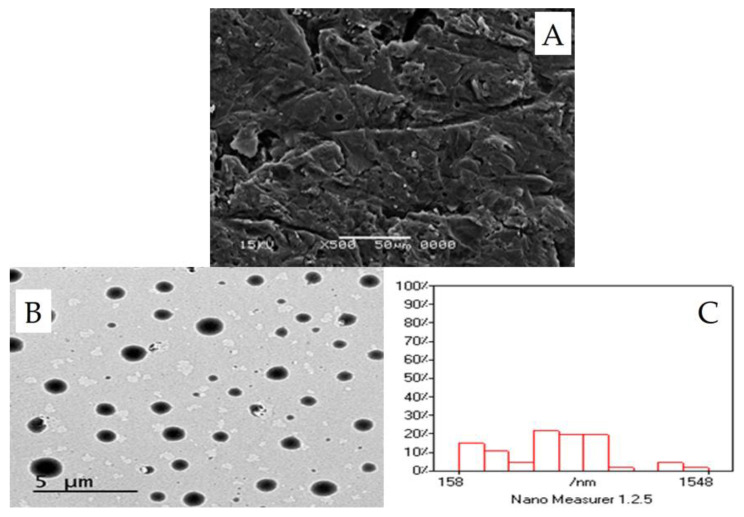
Morphological analysis of the CLX-IPP powder and the formed vesicles of the optimized formulation after hydration. (**A**) Scanning electron micrograph of the prepared powder. (**B**) Transmission electron micrograph showing the formed vesicles after hydration. (**C**) Analysis of the TEM image by Nano Measurer^®^ software.

The SEM of the lactose-based powder of the optimized formulation is shown in Figure 4A. As seen from the figure, the powder appears to have a smooth surface, while in the literature, lactose itself displays an irregular surface with some sharp corners [53]. The smoother surface might be due to the coating of the lactose surface with span 40 and cetyl alcohol. Blazek-Welsh and Rhodes presented an explanation for the influence of the coating process on the surface characteristics of carrier particles [54]. They suggested that the levelness and smoothness of the surface of lactose-based powder particles to be due to the packing effect of span 40 on the fine structures located on the surface of the lactose as carrier, where much thicker layers of span 40 were expected to be deposited at different points of deep invaginations. They have also claimed that there was brief dissolution of some surface molecules of the carrier, in particular, thin or sharp features, in the solvent mixture sprayed onto the carrier surface. When the solvent mixture was evaporated, the dissolved lactose surface molecules, as well as other components in the solvent mixture, were recrystallized onto the new surface. Consecutively, these effects evidently removed some of the crystalline fine structures of the carrier powder, making the surface of the lactose-based powder appear more even and smoother [53]. 

### 3.7. Morphology of the Optimized CLX-Loaded Nanovesicles Using TEM

The TEM of the optimized formulation is shown in Figure 4B, from the figure, it is clear that the obtained vesicles are completely spherical in shape, homogenous and uniform in size. They are present as discrete entities with defined boundaries. The size analysis of the TEM image by Nano Measurer software revealed that the vesicular size and distribution were comparable to the values obtained by the dynamic light scattering measurement. The vesicles were homogenous in size with narrow size distribution (Figure 4C).

### 3.8. In-Vitro Release of CLX from the Optimized Formulation

**Figure 5:**
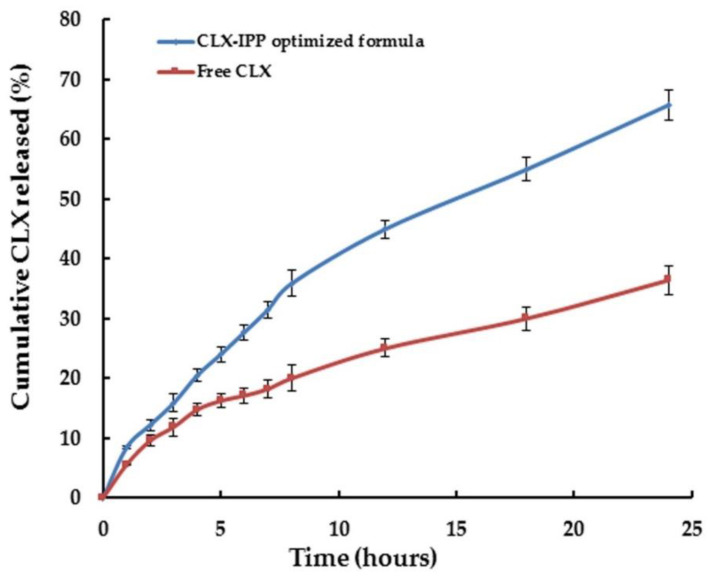
In-vitro release of free celecoxib (CLX) and the optimized formulation (CLX-IPP) in the dissolution medium (pH 7.4) containing 1% (*w*/*v*) sodium lauryl sulfate for 24 h.

The in-vitro release of CLX from the optimized CLX-IPP formulation compared to the pure drug was measured in release medium of phosphate-buffered saline pH 7.4 with 1% SLS. The release data were plotted in Figure 5. The addition of SLS was to maintain the sink condition, as according to Nasr, the aqueous solubility of CLX is only 3–7 μg/mL, while in 1% SLS phosphate-buffered its solubility was 106 μg/mL [27]. As seen from the figure, higher concentrations were released from CLX-IPP compared to the pure CLX. The optimized formulation achieved 65.73% cumulative release after 24 h, compared to only 36.41% of the free un-entrapped CLX. The higher amount released was due to the presence of span 40-based nanovesicles. The presence of cetyl alcohol led to the prolonged time dependent release shown in the CLX-IPP release pattern. Bandyopadhyay and Johnson mentioned that using cetyl alcohol as a carrier for span-based niosomes exhibits controlled in-vitro release of 5(6)-carboxyfluorescein dye as it released ∼20% of the encapsulated dye after 6 h [55]. The optimized formulation achieved a similar release percentage of CLX over the same time period (27.72%). Auda et al., found that the in-vitro release of CLX from poloxamer-based niosomal gel was about 72% after 12 h [56]. The presence of cetyl alcohol as a vesicle stabilizer might hinder the release of CLX for a prolonged period.

### 3.9. In-Vitro Cytotoxicity Study of the CLX-IPP Optimized Formula Using MTT-Assay

**Figure 6:**
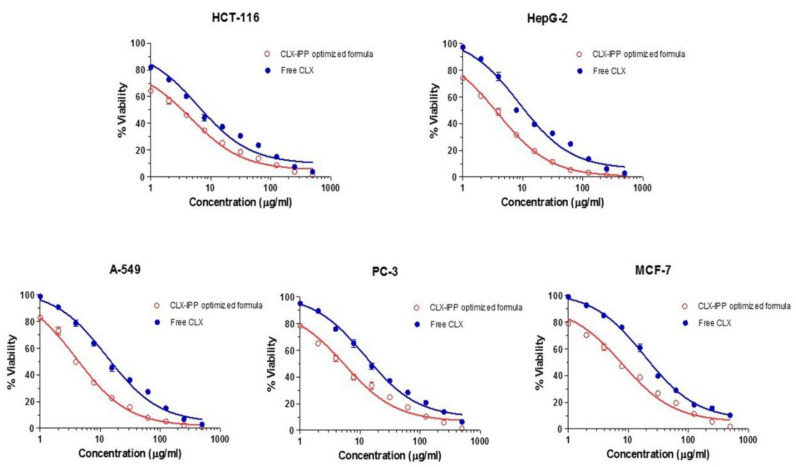
Antiproliferative effect of CLX-IPP optimized formula and free CLX on HCT-116, HepG-2, A-549, PC-3 and MCF-7 cell lines after 48 h exposure, as observed by MTT assay. Data on *x*-axis (concentration in ug/mL) are represented in logarithmic scale. Abbreviations: CLX-IPP, celecoxib in-situ provesicular powder; CLX, celecoxib; HCT-116, colon cancer cell line; HepG-2, liver cell line; A-549, lung cancer cell line; PC-3, prostate cancer cell line; MCF-7, breast cancer cell line.

We expected that nanoencapsulation would improve the biological effect of CLX; therefore, to verify this, the effects of free CLX and their nanoprototypes (CLX-IPP) were compared in five different cancer cell lines. It was found that the CLX-IPP optimized formula inhibited cancer cell growth to a greater extent than its free counter-part in all investigated cancer cell lines as shown in Figure 6. The cytotoxicity study results reveal that CLX had an anti-proliferative effect on HCT-116 colon cancer cell line with an IC_50_ of 6.42 µg/mL followed by HepG-2 liver cell line with an IC_50_ of 8.61 µg/mL then A-549 lung cancer cell line of 13.58 µg/mL, after that PC-3 prostate cancer cell line with an IC_50_ of 14.7 µg/mL and the lowest one was on the MCF-7 breast cancer cell line with an IC_50_ of 23.68 µg/mL. Accordingly, these data show the great cytotoxic effect of CLX on the previously mentioned cancer cells, which could be due to it being a selective COX-2 inhibitor in which COX-2 is overexpressed in different tumor cells and plays an important role in tumorigenesis as reported earlier [49,57]. In addition, it is supposed to have many probable antitumor mechanisms that are COX-2-independent, including prevention of proliferation, initiation of apoptosis, immunoregulation, regulation of tumor microenvironment and an antiangiogenic effect as stated by other researchers [3,58,59]. On the other hand, the optimized formula scored IC_50_ values of 3.30 µg/mL, 3.82 µg/mL, 4.001 µg/mL, 5.03 µg/mL and 7.01 µg/mL in HCT-116, HepG-2, A-549, PC-3 and MCF-7 cancer cell lines, respectively. Consequently, these results are of the highest importance since the CLX-IPP optimized formula showed a more cytotoxic effect than the free CLX; in which the IC_50_ values were reduced by two-fold for colon cancer, 2.2-fold for liver cancer, 3.39-fold for lung cancer, 2.9-fold for prostate cancer and 3.37-fold for breast cancer in comparison to the free CLX. The superiority of the in-situ provesicles could be due to their small size, which permits better cellular uptake and cell internalization, which leads to intracellular release of CLX, and hence improves anticancer activity. 

### 3.10. Pharmacokinetic Analysis of the Optimized CLX-IPP Formula Compared to Market Product in Rabbits

**Figure 7:**
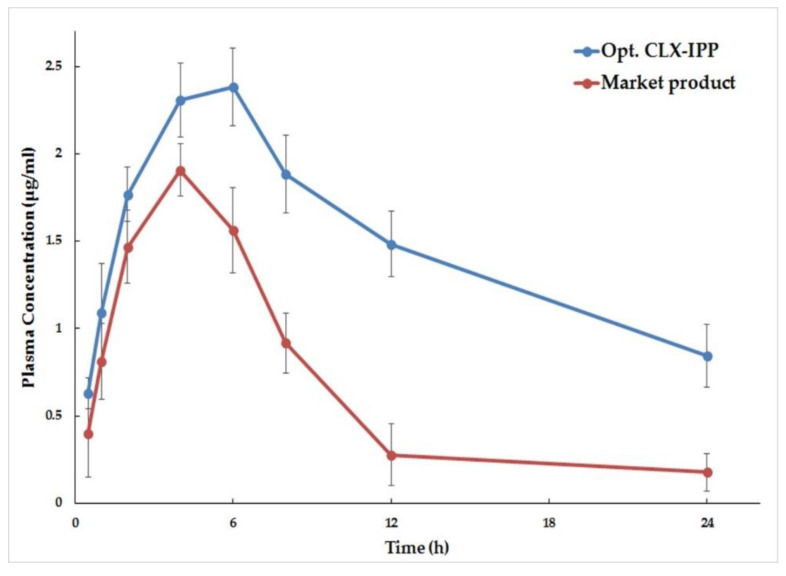
Plasma concentration–time profiles of celecoxib after oral administration of optimized CLX-IPP formula and market product Celebrex 100 mg capsule powder via per-oral route in rabbits (mean ± SD, *n* = 6).

To evaluate the pharmacokinetic parameters of CLX in the optimized IPP after oral administration, the rabbit plasma CLX concentrations were plotted versus time as shown in Figure 7. The pharmacokinetic parameters of the optimized CLX-IPP formulation and the market product are listed in Table 4. As shown from the table and the figure, it is clear that higher CLX plasma concentrations were achieved in the groups that were administered the tested CLX-IPP. The peak plasma concentration was found to be 2.38 ± 0.22 µg/mL compared to only 1.91 ± 0.15 µg/mL for the group that was administered the market product. The optimized CLX-IPP formulation showed a more sustained effect of the drug and the T_max_ was 6 h compared to 4 h for the market product. An increase in the AUC in the optimized formulation compared to the market product was seen, indicating higher oral bioavailability. The AUC_0–24 h_ obtained from the optimized formulation was found to be 35.748 ± 3.01 µg·h/mL, compared to 15.968 ± 2.18 µg·h/mL for the market product group. From the mentioned data, it was found that the relative bioavailability of the CLX-IPP formulation was increased by 220.87% compared to the marketed CLX product. The higher drug absorption might be attributed to the formulation of the drug into span 40-based nanovesicles. According to Jadon et al., higher extents of lipophilic drug absorption are achieved when formulated into span-based niosomes. This was explained as higher portions of drugs absorbed by transcytosis of M cells of Peyer’s patches at the intestinal lymphatic tissues [60]. The prolonged release of the drug in plasma was attributed of presence of cetyl alcohol as the fatty alcohols might increase the entrapment of CLX in the nanovesicles and hinder its release for a longer time. The pharmacokinetic study data in addition to the in-vitro cytotoxic study show that the optimized CLX-IPP can be considered as an optimum drug delivery nanosystem for using CLX in cancer therapy and it can modulate the drug release to achieve the best clinical results.

## 4. Conclusions

The findings of this study could support the assumption that using the COX-2 inhibitor CLX in a new repositioned use as an anticancer agent might be promising, as mentioned in studies that have evaluated its effect as a pure drug. The loading of CLX into novel cetyl alcohol/sorbitan monopalmitate nanovesicles resulted in an increase in CLX cytotoxic activity, in-vitro release, and improved its pharmacokinetic parameters. The prepared dry free flowing stable provesicular lactose-based powder is advantageous in increasing the stability and flowability of the pure drug when the formulation variables were optimized. The optimized formulation containing the drug was found to be a very promising delivery system for improving the bioavailability of CLX in-vivo. The optimized formulation has the potential of scaling up as the method of preparation is simple, rapid and involves a single step procedure. The possibility as well as the economic feasibility of the scaling up process should be investigated in future studies. 

## Figures and Tables

**Table 1:** Full factorial design (3^2^) used for preparation and optimization of celecoxib in-situ provesicular powders.

Independent Variables	Low	Mid	High
X1: Amount of Lipid	200	400	600
X2: Amount of Surfactant	200	300	400
**Dependent Variables (Responses)**	**Desirability Constrains**		
Y1: Particle size (PS)	Minimize		
Y2: Entrapment Efficiency (EE) %	Maximize		

**Table 2:** Composition of the prepared cetyl alcohol/sorbitan monopalmitate in-situ provesicular powders loaded with celecoxib.

Formula	Celecoxib Amount (mg)	Cetyl Alcohol Amount (mg)	Span 40 Amount (mg)	Spray Dried Lactose Amount (mg)
**F1**	200	200	200	1500
**F2**	200	300	200	1500
**F3**	200	400	200	1500
**F4**	200	200	400	1500
**F5**	200	300	400	1500
**F6**	200	400	400	1500
**F7**	200	200	600	1500
**F8**	200	300	600	1500
**F9**	200	400	600	1500

**Table 3:** Particle size (PS), polydispersity index (PDI), zeta potential (ZP) and entrapment efficiency (EE %) of CLX nanovesicles.

Formula	P.S	PDI	ZP	EE %
**F1**	621.2 ± 2.82	0.494 ± 0.019	−36.1 ± 0.67	92.67 ± 0.75
**F2**	522.5 ± 3.86	0.348 ± 0.047	−40.1 ± 0.96	94.51 ± 1.25
**F3**	461.0 ± 2.35	0.318 ± 0.011	−37.1 ± 1.36	88.56 ± 0.95
**F4**	447.5 ± 5.33	0.375 ± 0.007	−37.9 ± 1.32	91.50 ± 2.21
**F5**	520.1 ± 1.32	0.404 ± 0.023	−38.9 ± 1.72	92.49 ± 2.08
**F6**	429.4 ± 2.47	0.312 ± 0.033	−38.8 ± 1.33	97.39 ± 1.58
**F7**	381.8 ± 3.90	0.481 ± 0.072	−36.8 ± 1.78	95.61 ± 1.86
**F8**	389.4 ± 2.53	0.466 ± 0.025	−39.3 ± 0.75	95.20 ± 2.24
**F9**	351.7 ± 1.76	0.302 ± 0.046	−41.3 ± 0.90	97.53 ± 0.84

**Table 4:** Pharmacokinetic data from the curve fitting of in-vivo rabbit plasma data after peroral administration of the market product capsule’s powder and the optimized formulation (each equivalent to 10 mg/kg celecoxib) (*n* = 6) with SD.

Parameter	Celebrex 100 mg Caps	Opt. CLX-IPP
C_max_ (µg/mL)	1.91 ± 0.15	2.38 * ± 0.22
AUC_0–24_ (h·µg/mL)	15.968 ± 2.18	35.748 * ± 3.01
T_max_ (h)	4	6 *
T_1/2_ (h)	6.186 ± 0.17	14 * ± 0.25
K_el_ (h^−1^)	0.112 ± 0.018	0.0495 * ± 0.023
MRT (h)	9.509 ± 0.24	12.147 * ± 0.19

Abbreviations: (AUC) the area under the curve, (MRT) mean residence time, (SD) the standard deviation. * Significant difference (*p* < 0.05).
